# A Generalized NMF-Based Method for Analyzing Time-Resolved Spectroscopic Data

**DOI:** 10.1021/acs.jpca.6c00974

**Published:** 2026-05-11

**Authors:** Elizaveta Kobeleva, Surahit Chewle, Marius Horch, Marcus Weber

**Affiliations:** † Department of Physics, Ultrafast Dynamics in Catalysis, 9166 Freie Universität Berlin, Arnimallee 14, 14195 Berlin, Germany; ‡ Zuse Institute Berlin, Takustraße 7, 14195 Berlin, Germany

## Abstract

Time-resolved spectroscopy is a widely used tool for the investigation of physical and chemical processes. Analysis of the results is often challenging due to the inherent complexity of the data, encoding the chemical nature and time evolution of multiple species involved in the reaction. Many existing analytical methods are unsatisfactory as they introduce bias by relying on unjustified mathematical or mechanistic assumptions about the studied process. Here, we introduce a generalized analytical strategy based on non-negative matrix factorization. The methodology builds on a bottom-up model-free approach, in which physically grounded mathematical constraints can be introduced by active choice, allowing for an unbiased analysis of complex time series of spectroscopic data. The strength of this strategy is demonstrated by successful deconvolution of synthetic data mimicking different types of chemical reactions and typical challenges encountered in time-resolved Raman spectroscopy.

## Graphical abstract

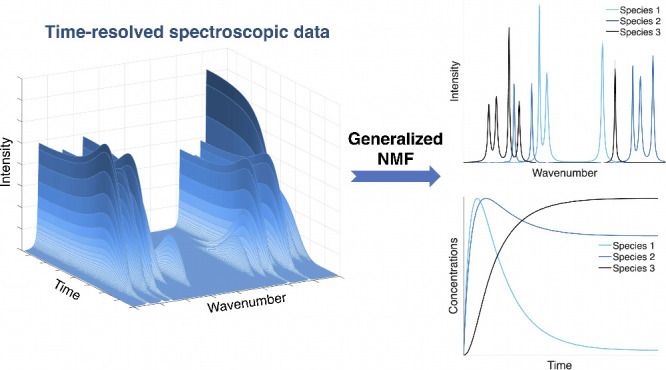

## 1 Introduction

Time-resolved spectroscopy is crucial for investigating a wide range of physical and chemical processes. Well-established vibrational methods, such as (resonance) Raman spectroscopy, probe vibrational modes, enabling the tracking of structural changes associated with physical process and chemical reactions in real time.^1,2^ However, data from time-resolved Raman (or other spectroscopic) experiments is typically complex, and specific chemical information is difficult to extract. Each Raman spectrum is a superposition of the spectral signatures of the chemical species involved in the reaction. The relative contribution of each species to the overall spectrum changes with time, allowing, in principle, extracting the time evolution of each component from one time-resolved experiment. Depending on the system under study, however, analysis of the data is often complicated by spectral similarity of different species and their different levels of resonance enhancement.^3^

Manual deconvolution of such data requires preliminary chemical knowledge of the system or assumptions about the studied process, which unavoidably introduces human bias and might lead to misinterpretation of the data. In addition, (resonant) scattering cross sections are hard to predict even for known chemical systems. Contrarily, approaches that address this issue from a purely mathematical perspective allow for an unbiased analysis of such data. Several of these techniques are based on the factorization of a matrix representing the time- and frequency-resolved data.^4^ However, obtaining a unique solution of the problem at hand typically requires rigorous mathematical assumptions about the properties of the data matrix, some of which are detailed below. This leads to the potential introduction of mathematical bias, which may pose even bigger problems than human bias, as the mathematical assumptions and constraints introduced in the specific method might be nonphysical and unknown to many users. In this work we will describe an alternative analytical tool based on non-negative matrix factorization (NMF), which builds on a bottom-up model-free approach, where physically grounded mathematical constraints can be included by active choice.

The article is organized as follows: In section 2.1, the basic NMF method is introduced, and some examples of known NMF-based algorithms are discussed. In sections 2.2 and 2.3, practical problems of mathematical constraints typically used in these methods are described, and an alternative strategy is suggested. In section 2.4, the algorithm for a generalized NMF-based matrix factorization method is introduced. In section 2.5, the performance of the algorithm is demonstrated for different types of data. In section 3, a discussion and outlook is provided.

## 2 Methodology and Results

### 2.1 Mathematical Background

Matrix factorization is a dimensionality reduction technique that decomposes a matrix into the product of two or more matrices. The general aim of NMF is to transform the non-negative data matrix into two non-negative submatrices. This approach is useful for analyzing data matrices with non-negative entries, as obtained in many spectroscopic experiments, where absolute absorption, emission, or scattering is recorded. In particular, for Raman spectroscopy, NMF is an applicable factorization tool, as demonstrated previously.^5,6^ For the mentioned techniques, the time evolution of signals, reflecting concentration profiles of chemical species, cannot be negative as well, so the main requirement of non-negativity of the input matrix is valid.^4,7^

A scheme of the NMF processing is shown in Figure 1. Let us represent the time-resolved spectroscopic data as an input data matrix **M** with dimension *m* × *n*, where the *m* correspond to the wavenumber elements and the *n* corresponds to time points at which the spectra were recorded. Then, each entry of the data matrix represents the intensity of the spectroscopic signal at a specific wavenumber and a specific point in time. This way, each column contains a spectrum (intensity as a function of wavenumber) at a specific time point, and each row contains the time evolution of intensity at a specific wavenumber. During factorization, we seek to approximate **M** as a product of two non-negative submatrices **W** and **χ**:

**Figure 1:**
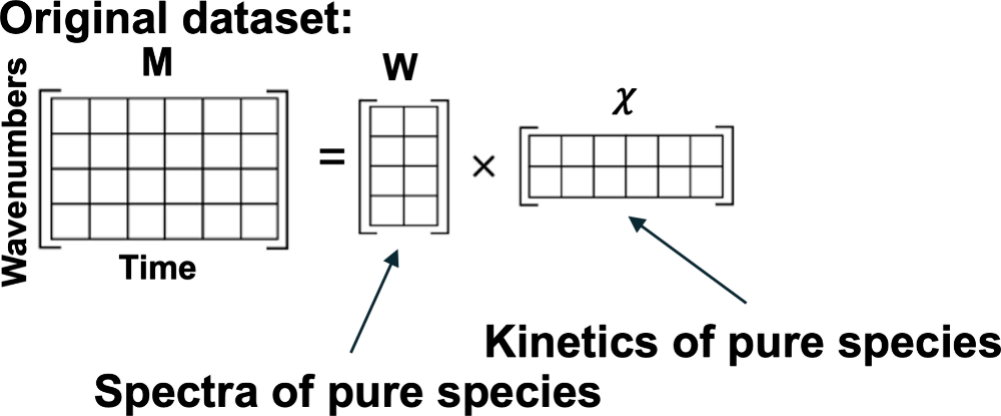
Schematic representation of the separation of a data matrix into two submatrices via NMF.

**M** ≈ **Wχ**

For a number of species *r*, the matrix **W** is a *m* × *r* matrix containing pure spectra of individual species as columns, while the matrix **χ** is an *r* × *n* matrix, containing the kinetics of the species as rows.

Without further constraints, the NMF problem is ill-defined and its solutions are not unique. Therefore, the NMF problem typically converts into an optimization problem that can be solved with an objective function. This objective function eventually constrains the output data to specific solutions based on physical or mathematical assumptions. Thus, the NMF algorithm searches for non-negative matrices **W** and **χ**, such that **Wχ** is not only close to the original matrix **M** but also satisfies further conditions defined by the objective function as much as possible. While most of the NMF algorithms search for approximate solutions of **W** and **χ**, some of them find exact solutions for **M** = **Wχ** by using inherently different optimization strategies. For example, by altering just the first matrix **W**, the second matrix **χ** can be recalculated from the optimized values of the first one as **χ** = **W** ^–1^ **M**.^6,7^

NMF has become a popular tool in the analysis of, e.g., spectroscopic time series and molecular dynamics trajectories. In the following, we will give an overview of the general workflow and most significant implementations^5,6,8^ as a basis for illustrating open challenges in the application of NMF to such problems.

The NMF approach generally requires an initialization based on the number of species to be considered. Singular value decomposition (SVD) is another common method of matrix factorization, which can be used for that purpose. SVD decomposes any real matrix into orthogonal submatrices. The SVD solution always exists and its singular values are unique, which makes it applicable to any spectroscopic time-resolved data. However, no additional constraints, such as non-negativity, can be introduced to its solution. Thus, the applicability of SVD is quite limited. While the occurrence of negative entries might not be a problem for some spectroscopic methods, the general approach of factorization into orthogonal submatrices is usually not physically sensible, as the assumption of orthogonality across both spectra and kinetics as well as wavenumber and time elements is generally not justified. For example, if spectroscopic data includes information from similar chemical species, their spectral signatures are not expected to be orthogonal. Nevertheless, for its simplicity and wide applicability, SVD is often used as an initialization tool within the NMF method.^9^ SVD is especially useful for determining the number of significant components in the data, which is needed for NMF-based analyses.

A typical workflow for analyzing spectroscopic data with NMF looks as follows:

*Step 1.* Acquire the spectroscopic data matrix **M**.

*Step 2.* Perform SVD analysis: Inspect singular values and determine approximate number of meaningful components (e.g., by a clear drop-off in singular values).

*Step 3.* Select the number of chemical or physical components *r* for NMF based on SVD insights.

*Step 4.* Apply the NMF approach of choice with rank *r*.

*Step 5.* Optimize values of the objective function with given constraints.

*Step 6.* Recover submatrices of spectra **W** and concentration profiles **χ**.

Existing NMF-based methods for the separation of time-resolved spectroscopic data differ mainly by the optimization routine and the optimization criteria. Common algorithms, the successive projection algorithm and the vertex component analysis, could be used to generate solutions for the matrix decomposition methods geometrically.

The successive projection algorithm represents values of the initial matrix **M** as coordinates of points on an *r*-dimensional simplex and then assumes that pure species correspond to the coordinates of the vertices of that simplex. The algorithm searches for those vertices, starting from the assumption that one of them corresponds to the column of **M** with the largest Euclidean norm. This is a very fast and universal algorithm. However, it applies rigid assumptions with no physical context and does not regard any optimality constraint.^10^ This successive projection algorithm has therefore been extended in the related successive nonnegative projection algorithm, which introduces the non-negativity constraint in the decomposition.^11^ Based on this, a separable NMF method for time-resolved spectroscopic data was described.^5^ Here, the successive nonnegative projection algorithm was applied to calculate the kinetic submatrix **χ**, while the spectra submatrix **W** was computed based on values of the original matrix **M** and the obtained kinetic submatrix via the minimization criterion $∥M-w_{\chi }∥$ $\;\overset{!}{=}\;$ min. In this approach, the separability of the component spectra was considered as a key constraint, meaning that each spectrum has at least one nonoverlapping contribution.^12^

Vertex component analysis, on the other hand, tries to approach the simplex optimization problem without the assumption of separability. However, it relies on preliminary knowledge of the data. Here, the columns of **M**, containing spectra at specific time points, that are closest to the vertices of the optimal simplex are expected to be known, so that there is no need to iteratively search for them. In other words, the spectra of the pure individual components involved in the process to be studied are known, at least approximately. While vertex component analysis is a relatively simple and reliable method, in practice, this condition might be even more problematic as it requires that the experimental data includes nearly pure spectra of all extractable species, meaning that at certain time points all species reach relative concentrations of 1, which is not always experimentally possible.^13,14^

Another example of geometrical optimization related to the previous two algorithms is PCCA+.^15,16^ PCCA+ solves an optimization problem to find the smallest simplex including the data points. The algorithm is implemented in such a way that an initial, nonoptimal simplex is generated and then a local optimization approach adjusts this simplex until reaching a local minimum of the objective function. In principle, the optimization criterion of the smallest simplex is not physically motivated for the general spectroscopic problem which means that PCCA+ might lead to a nonphysical decomposition of **M**.

While the outlined NMF implementations proved to be mathematically effective and robust, their validity with respect to physical and chemical problems is not thoroughly substantiated, which limits their applicability to specific cases. In the current account, we therefore evaluate constraints typically used in NMF methods in order to formulate a generalized and adaptable NMF strategy that limits both human and mathematical bias.

### 2.2 Typical Constraints in NMF-Based Methods

Previously introduced NMF-based methods often rely on sophisticated mathematical algorithms. Efficient application of such optimization algorithms requires the experimental data to be preprocessed, as detailed in the following, which limits the general applicability of these algorithms. In addition, preprocessing might distort the data and introduce the risk of misinterpretation even before analysis starts.

(1) Normalization is among the most common problematic requirements in typical NMF implementations. Matrix optimization methods commonly require the original data matrix to be row-wise and/or column-wise normalized before optimization can be performed. In this way, matrices can be sorted and clustered easier and more efficiently. In terms of experimental data, this would mean that both spectra and kinetics should be normalized, which typically introduces serious distortion of the data. For instance, in a typical time-resolved Raman experiment monitoring a physical or chemical process, some species (intermediates or components) could be undetectable, e.g. due to a low Raman scattering cross section. Assumption of visibility of all significant intermediate states may introduce severe misinterpretation. Specifically, the resonance Raman effect may contribute to significant differences in spectral intensities of chemical components of equal concentration. Therefore, normalization of such data is not physically sensible.

(2) Another requirement that is key to the success of some established NMF strategies is separability of the data. In the current context, separability means that the solution of the matrix factorization is requested to have nonoverlapping spectral components with nonidentical time traces. Solving the separability problem, the algorithm would look for a solution yielding “the most different” components, a simple mathematical interpretation of which would be orthogonality. Examples of this are SVD-based methods and others that use orthogonality as one of the optimization criteria. However, for real-world problems, this constraint appears to be too strict and to compete with the non-negativity condition. In addition, the orthogonality criterion is hard to interpret and satisfy for spectroscopic data in general. Realistic experimental cases often include structurally and therefore spectrally similar components, which would not obey such constraint.

(3) More constraints can arise from mechanistic assumptions regarding the studied process. To describe kinetics of a chemical or physical process mathematically, a Markov process model is often used in established NMF analyses as it allows to predict the evolution of the system based only on its current state.^17,18^ In general, Markovian behavior might be hard to confirm for real world chemical or physical problems. Even though the implied lack of memory is typically fulfilled, especially for chemical processes, the definition of the system and its states is not trivial. For reactions where molecular species undergo dissociation or association, the Markovian states of the system are *not* identical with the molecular species under study, so that spectroscopic and kinetic properties *cannot* be interpreted in a chemical sense. In addition, the current state of the studied system should be known, which is not always the case, due to possible occurrence of intermediates that are spectroscopically undetectable or indistinguishable. Since the actual reaction mechanism is typically not known (but rather to be determined by the time-resolved experiment), the application of other kinetic models within the analysis is highly problematic as well.

### 2.3 Reduction of Optimization Constraints

Strict mathematical constraints are obligatory requirements for the application of most NMF algorithms. However, taking the above arguments into consideration, they can lead to serious problems or severe limitations in the applicability of the NMF method when analyzing time-resolved spectroscopic data. Given the problems associated with these constraints and the preprocessing of data, we suggest a ground-up approach for the NMF-based algorithm. We formulate it in a way that reduces nonphysical constraints to a minimum. On the one hand, this approach eliminates analysis-induced misinterpretations; on the other hand, it provides a simplified and generalized method, applicable to a greater range of data and scientific problems.

Conceptually, we propose a customizable bottom-up approach for setting up the objective function. In the basic configuration, the number of optimization criteria are minimized to avoid any nonphysical constraints. Additional criteria that are justified and applicable to a specific problem can then be introduced to narrow down the number of solutions in a manner that is firmly grounded in physical and chemical concepts.

The initial goal is the removal of all constraints except for the most general ones, in particular non-negativity of both extracted submatrices corresponding to pure spectra and concentration traces of the individual components. For effective optimization, the additional constraint of spectra separability is also included. However, the latter is problem-specific and can be altered or removed in cases- where different conditions are relevant. In contrast to the other methods summarized above, our approach is formulated without restrictions on concentration traces, such as a row sum of 1 or the assumption of a Markov process. Notably, this means that the only condition applied to concentrations is non-negativity. As a consequence, the factorization employed here is essentially model-free and therefore independent from preconceived notions about the studied process or unjustified mathematical constraints. The optimization of the submatrices is performed primarily with respect to the spectra. In our opinion, such an approach is more sensible as the appearance of component spectra is normally easier to evaluate than the component concentration profiles of a complex reaction. For instance, Raman spectra and related quantities, for example isotopic shifts, can be calculated by electronic-structure methods like density functional theory, which allows the validation of NMF-predicted spectra and the structural identification of the associated intermediates. Finally, to follow the simplification tactic we use, an unambiguous and robust least-squares optimization method for minimizing the objective function is employed. In the current case, the objective function is set up as a linear combination of variables corresponding to chosen optimization criteria, whose values can be chosen to be maximized or minimized. This set up also implies that the different criteria can be weighted based on an informed user choice.

The algorithm is organized as a two-step process: in the first step, the main objective function is optimized, and in the second step, a customizable objective function with additional problem-specific constraints is optimized. The algorithm is implemented in MATLAB, but could be easily transferred to the programming environment of choice.

### 2.4 Code for Analyzing Time-Resolved Spectroscopic Data

In the following, the implemented approach introduced in the previous section will be described in detail. The pseudocode for the first step of optimizing the main objective function is outlined as Algorithm 1 and described subsequently.

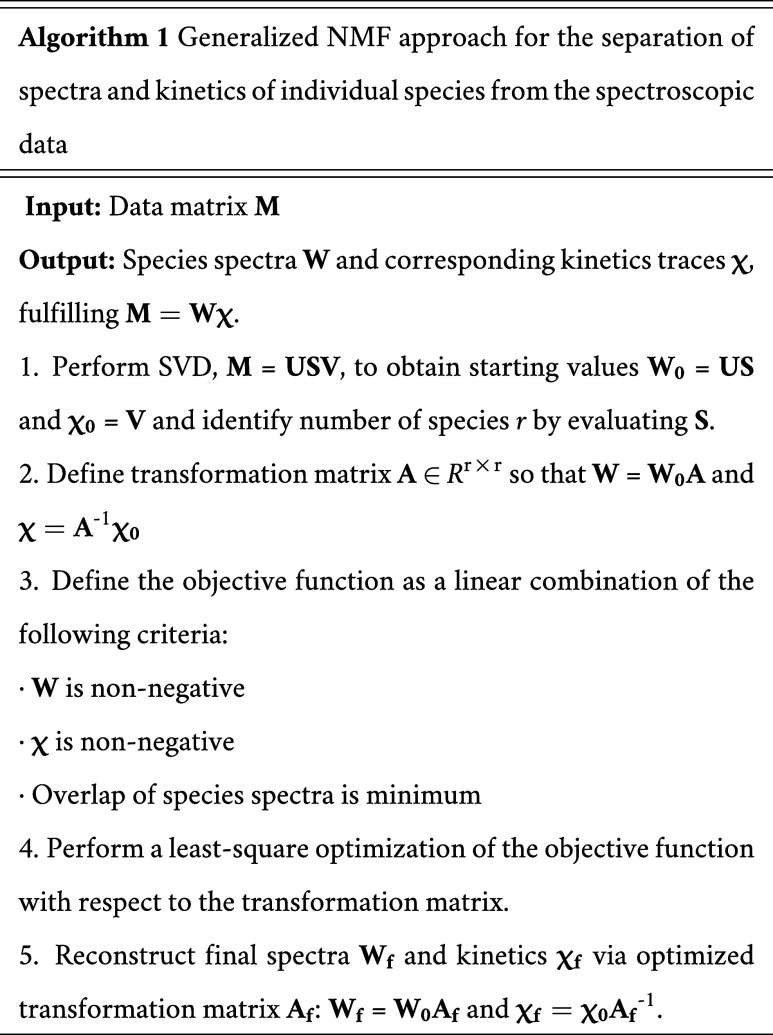



While our method does not require preprocessing of data, we found that basic procedures such as baseline correction and filtering of noise- can improve performance significantly, for example if a fluorescence background is present in Raman spectra. Of note, such procedures are often employed in the analysis of spectroscopic data. They are neither dictated by the mathematical framework of the NMF approach nor prone to distort the data, if utilized properly. However, the decision about such preprocessing should be made on an individual basis, in particular for data with low signal-to-noise ratio and weak components present. If justified, we make use of the *datasmooth* function in MATLAB for filtering noise via the Savitzky-Golay method and subtract a baseline via a spline fitting function. Without these steps, the algorithm would separate the background as a separate species, which would increase the total number of species, thereby unnecessarily limiting the efficiency of the NMF procedure.

*Step 1.* To obtain initial values of spectra **W** and kinetics **χ** matrices, SVD (**M** = **USV**) is performed. Here **U** and **V** are the two orthogonal submatrices and **S** is a diagonal matrix containing the singular values of **M**. This method is chosen as a simple one-step way to automatically obtain **W**_**0**_ = **US** and **χ**_**0**_ = **V** matrices that obey the equation **W**_**0**_ **χ**_**0**_ = **USV** = **M** exactly if the rank of the SVD matches the actual number of components considered in the analysis. This is achieved as follows: Initially, SVD is performed for 10 components, as the expected number of species in the data is clearly lower. Thus, if the matrix **M** has dimension *m* × *n*, then **W**_**0**_ has dimension *m* × 10 and **χ**_**0**_ has dimension 10 × *n*. For this purpose, the MATLAB function *svds*, which allows to specify the number of components, is used. The user can then estimate the real number of components *r* based on the singular values in matrix **S** and return that estimated number to the algorithm for further processing. This way, initial matrices **W**_**0**_ and **χ**_**0**_ are truncated to the chosen dimensions of *m* × *r* and *r* × *n*, respectively.

We like to stress that estimating the number of components from the SVD is the only step in the basic implementation of our approach that requires a user decision and, as such, could be considered a restriction to the idea of a model-free approach. While this potential source of human bias could also be removed by using a mathematical criterion for the determination of the rank, we feel that, without prior knowledge of the problem at hand, it is more advisible to rely on a user-based decision and systematic testing of the impact of the rank. The latter can be achieved by simply varying the determined number of components in the subsequent analysis, thereby exploring the impact of the choice made. This might be advisible, in particular, in cases where weakly contributing components are expected.

*Step 2.* In the next step, a transformation matrix **A** with size *r* × *r* is defined, initially filled with random values. This transformation matrix is optimized according to the criteria of the objective function and used to determine the target matrices **W** and **χ** that properly describe the studied problem. The optimization function and criteria are formulated in the next step, and the values of the transformation matrix and, correspondingly, of the matrices **W** and **χ** are then updated with every iteration of the optimization routine. Specifically, the target matrices are calculated as **W** = **W**_**0**_ **A** and **χ** = **A** ^–1^ **χ**_**0**_, so that the relation **Wχ** = **M** is satisfied automatically. The exact representation of the data matrix **M**, rather than an approximation, is one of the advantages of this NMF method compared to several previous ones.

*Step 3.* Optimization criteria are defined and evaluated based on the current value of the transformation matrix **A** and the corresponding values of target matrices **W** and **χ**. Penalty values with respect to each criterion are calculated as follows:

• **W** is not negative: *v*_1_ = *minimum*(**W**_**min**_)^2^– determine the smallest element of entire matrix **W**, and then ensure that it is as close to zero as possible, using its squared value as a penalty *v*_1_.

• **χ** is not negative: *v*_2_ = –*minimum*(**χ**_**min**_,0) – determine the smallest element of matrix **χ** and compare it with zero, discriminating specifically against negative values, so that the smallest among all negative values is used as penalty *v*_2_. In this way, we discriminate against all partially negative kinetics traces.

• Pure spectra overlap is minimal: *v*_3_ = ∥|*W*_*i*_|·|*W*_*j*_|∥ – the overlap between spectra of species *i* and *j*, is calculated as the norm of the scalar product of the two spectra. In practice, the overlap is evaluated for all pairs of spectra at once. This is done by calculating the product of the absolute value of the current spectra submatrix **W** with the absolute value of the transposed submatrix **W** ^**T**^ and then taking the norm. The resulting value is used as the penalty value *v*_3_. Taking the absolute values ensures that possible negative entries are also considered in the penalty. For datasets where overlap of the spectra is expected, this criterion can be softened by adding a weighting coefficient 0 ≤ *a*< 1 to the penalty value *v*_3‑*soft*_ = *a*·*v*_3_.

*Step 4.* The objective function is then formed as a simple sum of the individual penalty values: *v* = *v*_1_ + *v*_2_ + *v*_3_. This function is minimized via a least-squares optimization routine with respect to the value of the transformation matrix **A**. In MATLAB, the *fminsearch* routine is used as the optimization tool to find the minimum of the objective function by varying the values of matrix **A**. Optimizing the transformation matrix rather than the target matrices of spectra and concentration profiles, **W** and **χ**, is a common and efficient way to reduce the dimensionality of the problem. Starting from random values of the transformation matrix **A**, the target matrices **W** and **χ** are computed based on the penalty values for each criterion. The objective function is then minimized and the optimized entries of the matrix **A** are determined. This process is repeated for *n* iterations, with the new values of the objective function being compared with all the previous ones in each step, and the matrix **A**_**f**_ corresponding to the overall minimum of the objective function is chosen to set the final values of **W** and **χ**. The optimal number of iterations is chosen empirically as a compromise between successive improvement of the optimization and increasing computational time. The suggested default number of iterations for experimental data is *n* = 1000.

*Step 5.* After the optimized value of the transformation matrix **A**_**f**_ is defined, resulting spectra and kinetics are reconstructed as follows: **W**_**f**_ = **W**_**0**_ **A**_**f**_ and **χ**_**f**_ = **χ**_**0**_ **A**_**f**_^–**1**^. As our method works without normalization with respect to the maximum spectral intensity or the maximum concentration, postprocessing steps for representative visualization may take place afterward, as detailed below.

For difficult cases and those where prior information about the spectra is available, an additional optimization routine for the further improvement of the accuracy of the target matrices might be sensible. This optimization is realized by a different algorithm; however, it follows the same approach as the first one, just with a modified objective function and different optimization criteria. Instead of relying on basic criteria like the initial algorithm, the additional algorithm only contains problem-specific optimization criteria, which are necessary to resolve remaining inaccuracies after applying the initial algorithm. In the additional algorithm, optimization is not iterative, and the resulting values from the first algorithm are used as initial values. Based on these, the second algorithm searches for reoptimized values, within certain boundaries with respect to the resulting values from the first algorithm. This approach assumes that the results of the first algorithm are already very close to the true ones, with distances smaller than those set by the boundaries. Under these conditions, the true values can be found by searching for reoptimized values within those boundaries. The additional algorithm should be adjusted to each specific problem and sometimes to the specific outcome of the first algorithm.

In the following, we exemplarily describe a second algorithm, called Algorithm 2, for a dataset in which some of the species are spectrally well isolated from others, while integrated intensities of the species differ significantly. In the NMF analysis, this can result in a mixing of the most intense species with other less intense ones. Such a scenario is an important case for time-resolved resonance Raman experiments, in which intermediates with very different levels or resonance enhancement can occur. In such a case, we can select spectral ranges where contributions from some specific species are expected as well as those where contributions of some other specific species are not expected, formulating separable or partially separable optimization criteria.

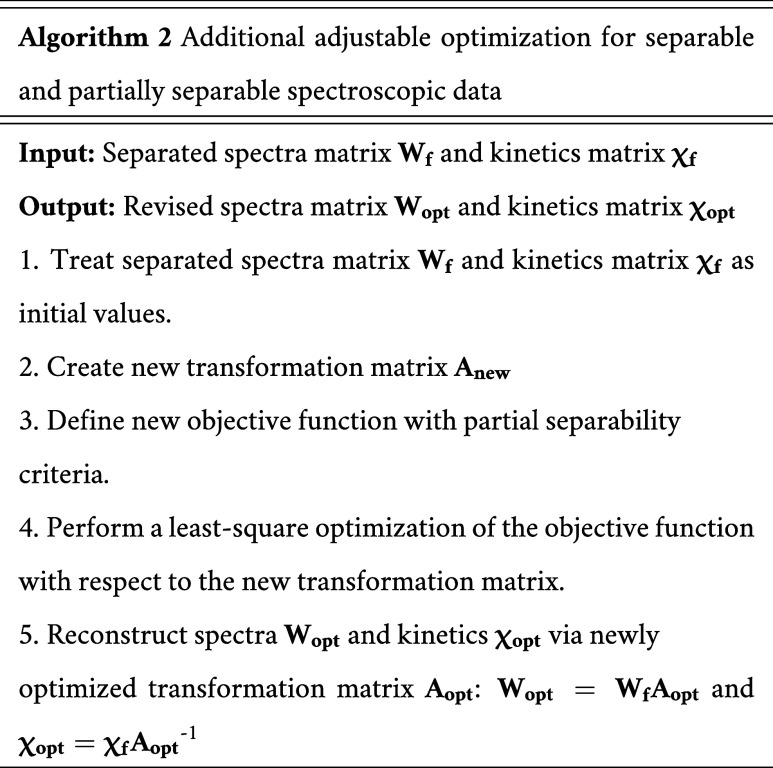



As the second algorithm follows the same method as the initial one, only new aspects regarding the optimization criteria are stated above and the minimization of the objective function are described in the following.

For *r* species let us define a spectral range from frequency *f* = *a* to *f* = *b* where spectral contributions from only *one* species *m* are expected. We define *S*_*i*_^*ab*^ as the spectral contribution of each species *i* to this range. The penalty value is then calculated as a sum of integrals of absolute intensities over the specific frequency range for all pure species spectra except species *m*: *v*_*m*_ = ∑_*i*=1,*i*≠*m*_^*r*^∫_*a*_^*b*^|*S*_*i*_^*ab*^(*f*)| d*f*. In addition, we also can define a spectral range from frequency *f* = *c* to *f* = *d* where no contributions from species *m* are expected: *S*_*i*_^*cd*^ is the spectral contribution of each species to this range, and the corresponding penalty value is *v*_*m*_^′^ = – ∑_*i*=1,*i*≠*m*_^*r*^∫_*c*_^*d*^|*S*_*i*_^*cd*^(*f*)| d*f*. Then, the overall objective function is defined as *v* = *v*_*m*_ + *v*_*m*_^′^.

The value of the objective function is minimized in MATLAB using *fminsearchbnd*, which performs a single-step search for values of the matrix **A** that correspond to the minimum of the objective function and lie within certain boundaries from the initial values.

### 2.5 Demonstration of Code Performance

In order to demonstrate the performance of the newly developed NMF approach, artificial datasets, modeling typical situations in time-resolved Raman spectroscopy, were generated. In contrast to experimental data, individual spectra and concentration profiles are known exactly, which allows evaluating the performance of the algorithm reliably. To illustrate its strength, we consider different kinds of kinetic scenarios. One of the datasets represents arbitrary time traces with uncorrelated kinetics, while another one simulates a first-order chemical reaction with branching. With these cases, we illustrate the applicability of our constraint-free approach to both types of data: one where no kinetic model is applicable, which would be a severe problem for the previously discussed algorithms, and one that follows a clear kinetic model but without prior knowledge about it included in the code.

The first artificial dataset consists of three independent spectral components represented by partly overlapping Lorentzian lines (spectral submatrix **W**). The time evolution of the intensity of these lines, indicating a change of the underlying populations (kinetic submatrix **χ**), was created manually as arbitrary time traces that do not reflect any kind of predefined kinetic model. As an input, the code receives the overall data matrix **M**, here with dimension 1000 × 172, constructed as **M** = **Wχ**, with the aim to recover the submatrices **W** and **χ** containing the spectra and kinetics of the individual components, respectively. As described above, the initial values for the submatrices are obtained from SVD in a first step. The user can then select the number of components based on the singular values, and in the current case the number of species is known to be 3. In the second step, the 3 × 3 transformation matrix **A**, filled with random values, is defined. The third step remains unchanged as the only optimization conditions are that the new resulting submatrices **W**′ and **χ**′ are non-negative and that overlap between the spectra is minimum. Then, in the fourth step, the values of matrix **A** are optimized iteratively with respect to those criteria using the *fminsearch* routine. Here, about 500 iterations were sufficient. In the fifth step, the final matrices **W** and **χ** are calculated. For this example, no additional optimization step was needed as almost exact results were found in the first step. In Figure 2, the resulting spectra and time traces are shown as solid lines. The true results (predefined spectra and time traces of the individual species) are shown as dashed lines. The accuracy of the employed approach is very high, so that original and reconstructed data overlap completely.

**Figure 2:**
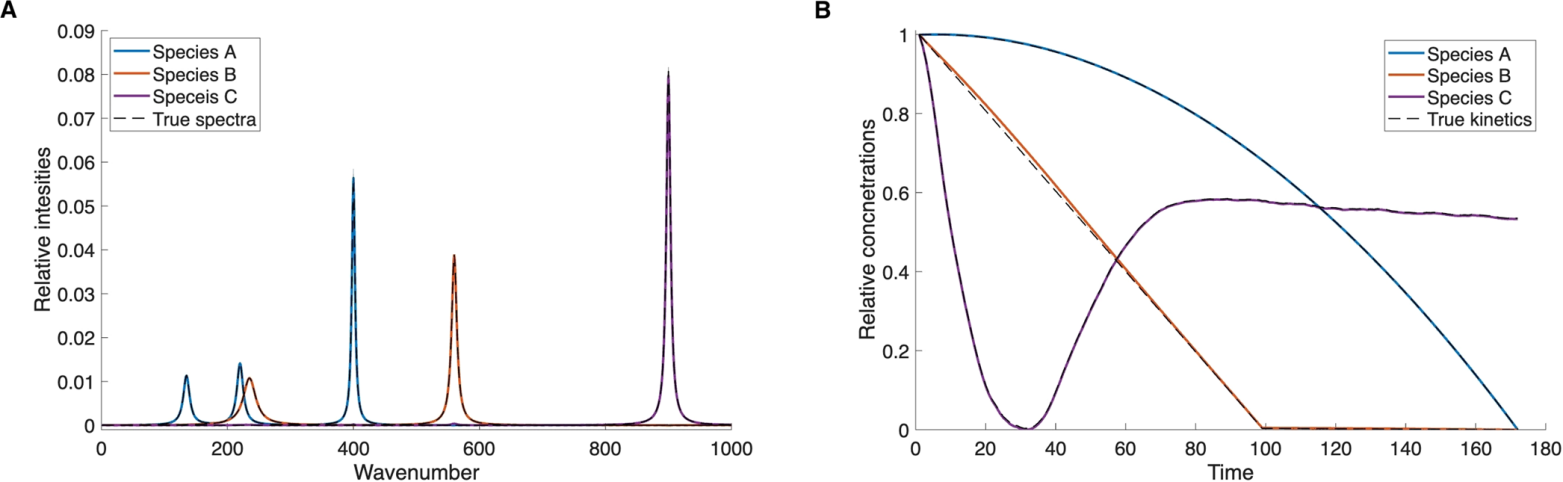
NMF-extracted results for artificial data with 3 species and uncorrelated kinetics. (A) Spectra of individual species. (B) Kinetics of individual species.

In Figure 3, the same example is shown, but with noise added to the constructed data matrix **M**. Also in this case, the NMF separation results are indistinguishable from the true spectra and concentration profiles. This illustrates that noisy data does not pose a problem to the algorithm, and it can work with this additional challenge without loss of accuracy.

**Figure 3:**
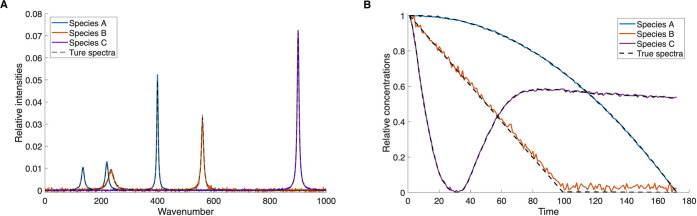
NMF-extracted results for artificial data with 3 species and uncorrelated kinetics. Noise has been added to the constructed data matrix **M**. (A) Spectra of individual species. (B) Kinetics of individual species.

Before analyzing the second artificial dataset, we will briefly discuss the postprocessing of results and their representation. Since the optimization method does not deal with the spectra and kinetic matrices **W** and **χ** directly, but only with the transformation matrix **A**, both resulting matrices are relative and differ from true values as

1 $$W_{\mathrm{result}}=W_{\mathrm{true}}\cdot C,\mathrm{and},\chi_{\mathrm{result}}=\frac{\chi_{\mathrm{true}}}{C}$$

In our method the product of the two submatrices is equal to the original matrix **M**, so that the resulting matrices fulfill the equation:

2 $$W_{\mathrm{result}}\chi_{\mathrm{result}}=\left(W_{\mathrm{true}}\cdot C\right)\left(\frac{\chi_{\mathrm{true}}}{C}\right)=W_{\mathrm{true}}\chi_{\mathrm{true}}=M$$

For visualization, we simply normalize spectra and kinetics in postprocessing. This way, and unlike using normalization in preprocessing, kinetic traces are not deformed during the analysis. In addition, the resulting matrices can be fitted to selected experimental values, e.g. the known maximum intensity of a certain spectroscopic line or the concentration of a certain species at a defined point in time. In this way, the entire matrices will be set to the true values.

For the second artificial dataset example, we created a system of differential rate equations corresponding to a branched process of first-order reactions that transforms species A into species D via two intermediates B and C:

Species A (Raman silent): $\frac{dI_{A}}{\mathrm{dt}}=-k_{1}I_{A}-k_{2}I_{A}$

Species B: $\frac{dI_{B}}{dt}=k_{1}I_{A}-k_{3}I_{B}$

Species C: $\frac{dI_{C}}{dt}=k_{2}I_{A}-k_{3}I_{C}$

Species D: $\frac{dI_{D}}{dt}=k_{3}I_{B}+k_{4}I_{D}$

where *k*_1_ = 0.05, *k*_2_ = 0.1, *k*_3_ = 0.01, *k*_4_ = 0.1 and *I*_A_(0) = 1, *I*_B_(0) = 0, *I*_C_(0) = 0, *I*_D_(0) = 0.

A kinetic scheme of this reaction is shown in Figure 4 on the left.

**Figure 4:**
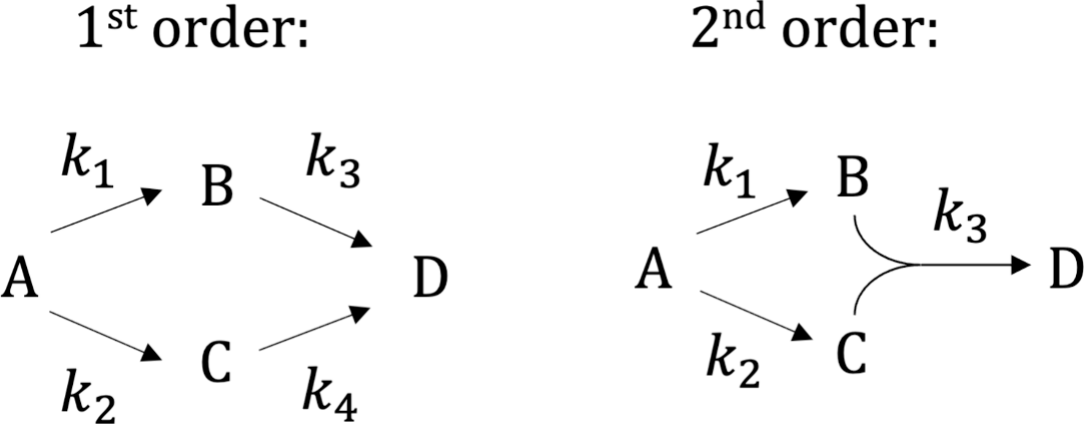
Kinetic schemes for the first and second order reactions.

The derived integrated rate laws were used to set the time traces of the components, while the spectra were represented by partly overlapping Lorentzian lines. We consider two cases here: In the first one, the dataset consists of 3 components: the two reaction intermediates B and C with very similar rate constants of formation and spectra and a product D that is spectrally and kinetically separated. We treat the educt A as a Raman-silent species. This case is important as it replicates experimental datasets, which were a motivation for developing this algorithm. In the next case, 4 components are considered, the 3 species above and the educt species, which is now included as Raman-active.

The 3-component dataset is separated nearly exactly, as shown in Figure 5. Even the very similar kinetic patterns of species B and C are extracted correctly. Once again, the true spectra and kinetics of the individual species are shown as dashed lines. To reach that result, the additional optimization step was necessary and formulated specifically for minimization of overlap of species B and C, according to the procedure described in section 5. This example shows that our algorithm is able to resolve a mathematically complicated problem with significant spectral and kinetic similarity within the framework of a specific kinetic mechanism. No preexisting knowledge about the reaction mechanism is required to reach that goal.

**Figure 5:**
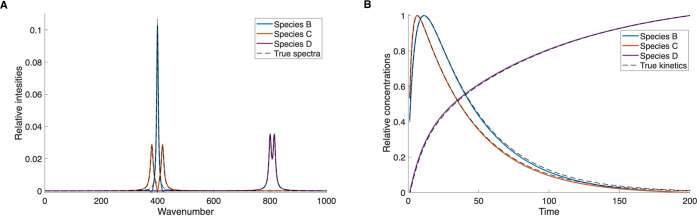
NMF-extracted results for artificial data with 3 species following a kinetic model of a branched reaction of first-order elementary steps. (A) Spectra of individual species. (B) Kinetics of individual species.

The second case of a 4-component system containing an observable educt species that is well separated in terms of spectral properties and time evolution is also resolved with sufficient accuracy. Results of the NMF separation are shown in Figure 6. However, we note a deviation in the spectrum of species C: the spectroscopic features of this species remain partly mixed with those of species B even after implementation of an additional optimization step as for the first dataset. The deviation in the spectrum also leads to an inaccuracy in the kinetic traces. While the general trend and rates of the kinetics traces are replicated correctly, some of the recovered kinetic components show visible deviation from the true traces. Apart from that, both spectra and kinetics of all species are extracted with acceptable accuracy, so that both the reaction mechanism and the spectral fingerprints of the involved compounds can be recovered.

**Figure 6:**
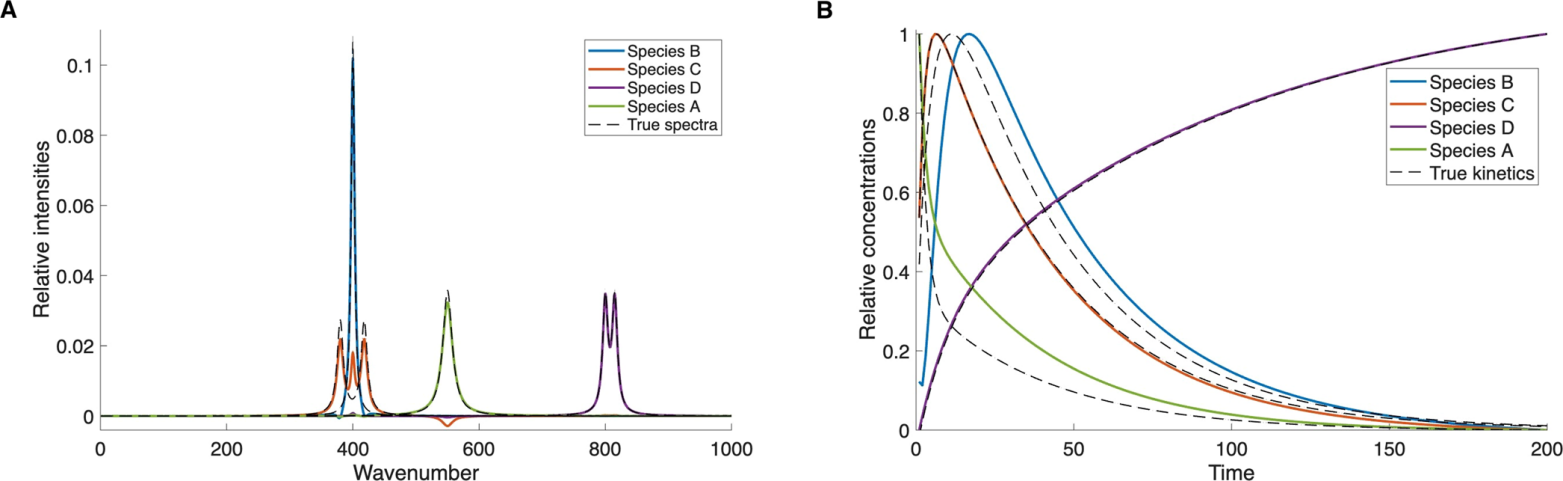
NMF-extracted results for artificial data with 4 species following a kinetic model of a branched reaction of first-order elementary steps. (A) Spectra of individual species. (B) Kinetics of individual species.

Comparison of the results obtained for the related 3-components and 4-components datasets allows identifying key factors that determine the accuracy of the algorithm performance. As noted above, the occurrence of spectrally and kinetically similar species does not pose a problem for the algorithm. However, an increased number of species may limit the overall accuracy of the results in a way that is not directly related to the complexity of the problem itself. We specifically note that the additionally included species in the 4-compound dataset is actually well separated, indicating that any inaccuracies in the results are related to the optimization procedure rather than the introduced NMF approach itself.

The next example is another artificially created dataset that models a second-order chemical reaction, with the corresponding kinetic scheme shown in Figure 4 on the right. Here, 3 components are considered. Again, the spectra were created by combining several Lorentzian lines, and the overlap between different species was kept low. For the time traces, we adjusted the previously used system of differential equations, so that it represent a Raman-silent educt species A that reacts into product species D through combination of the intermediate species B and C. This process is described by a new system of differential equations:

Species A (Raman silent): $\frac{dI_{A}}{dt}=-k_{1}I_{A}-k_{2}I_{A}$

Species B: $\frac{dI_{B}}{dt}=k_{1}I_{A}-k_{3}I_{B}I_{C}$

Species C: $\frac{dI_{C}}{dt}=k_{2}I_{A}-k_{3}I_{B}I_{C}$

Species D: $\frac{dI_{D}}{dt}=k_{3}I_{B}I_{C}$

where *k*_1_ = 0.05, *k*_2_ = 0.025, *k*_3_ = 0.1 and *I*_A_(0) = 1, *I*_B_(0) = 0, *I*_C_(0) = 0, *I*_D_(0) = 0.

This system was solved numerically and the solutions were used to describe the true time evolution of the species. The results of the NMF deconvolution are shown in Figure 7. Also in this case, the spectra and concentration traces are separated nearly completely, except for small negative artifacts in the spectrum of species D, and good overlap with the true spectra and kinetics, shown as dashed lines, is obtained. This illustrates an important capability of the model-free algorithm: Since there is no input or assumption regarding the physical or chemical nature of the investigated process, the order of reaction is irrelevant, and an increase in order does not affect the algorithm performance. In a more general sense, this means that the algorithm can treat various complex processes without preexisting knowledge on their mechanism. This is in sharp contrast to, e.g., global or target analyses,^19^ which require a solid understanding of the studied process to obtain meaningful results and avoid gross misinterpretation of the data. In this context, another key advantage of our model-free approach is the possibility to separate the identification of spectral components and their time evolution from the chemical or physical modeling of the studied process. The latter can be realized without interfering with the deconvolution of the data. Existing pieces of information may still be considered, if feasible, by design of the objective function, but no holistic model is required.

**Figure 7:**
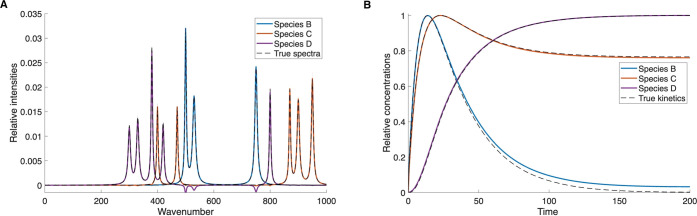
NMF-extracted results for artificial data with 3 species following a kinetic model of a branched reaction of first- and second-order elementary steps. (A) Spectra of individual species. (B) Kinetics of individual species.

## 3 Discussion and Conclusions

In conclusion, we have demonstrated a newly developed NMF-based analysis method for time-resolved spectroscopic data. The method is built as a generalized matrix factorization tool that is essentially model-free and independent from implicit nonphysical constraints during the optimization. Compared to existing NMF methods, our new method is significantly simpler and emphasizes a bottom-up approach that starts with a minimum number of constraints that can be adjusted by the user based on individual requirements and justified assumptions for a specific problem. This way, all choices made to obtain a unique and physically reasonable solution are traceable, and possible bias in the analysis can be minimized.

This study presents a conceptional work, which serves a basis for methodological solutions for a wide range of experimental problems within and beyond time-resolved spectroscopy. As an outlook, further advancements can be made depending on individual requirements, e.g., for specific experimental methods. This may include the reintroduction of tight boundary conditions and constraints where their application is justified and useful for improving performance. In addition, already introduced constraints might be reconsidered in order to extrapolate the method to other experimental techniques. This mainly applies to the non-negativity condition of the spectra, which limits application to spectroscopic data with negative components, as, e.g., obtained in difference spectroscopic approaches. In this context, we like to stress that the current method represents a generalized matrix factorization approach that does not necessarily depend on non-negativity. Besides that, the employed least-squares minimum search function used represents a simple and robust widely available optimization routine. The latter advantage, however, comes at the price of certain limitations. Changing to a more advanced optimization tool might be a useful measure to improve the method’s overall performance. On the one hand, this would minimize the computational time and increase accuracy, especially in cases where the number of required iterations is high. On the other hand, global optimization becomes inherently difficult for problems of high dimensionality, irrespective of the explicit implementation of the NMF approach. We expect that future developments regarding this aspect will further expand the applicability of the introduced approach to study complex processes with an increased number of intermediary species.

## Data Availability

The matlab code described in section 5 is available at https://zenodo.org/records/18517827 (DOI: 10.5281/zenodo.18517827).
